# The Use of Plant Growth-Promoting Bacteria to Prevent Nematode Damage to Plants

**DOI:** 10.3390/biology9110381

**Published:** 2020-11-07

**Authors:** Elisa Gamalero, Bernard R. Glick

**Affiliations:** 1 Dipartimento di Scienze e Innovazione Tecnologica, Università del Piemonte Orientale, Viale T. Michel 11, 15121 Alessandria, Italy; 2 Department of Biology, University of Waterloo, Waterloo, ON N2L 3G1, Canada; glick@uwaterloo.ca

**Keywords:** plant-parasitic nematodes, plant growth-promoting bacteria, biocontrol, plant protection

## Abstract

**Simple Summary:**

It has been estimated that 100 g of bulk soil can host about 2000–4000 nematodes and this amount is increased 5-fold in the rhizosphere. A certain number of these nematodes are pathogenic for plants and cause yield and economic losses. Application of chemical nematicides is the most common method used to reduce nematode populations, but these chemicals can have a negative impact on both the environment and human health. Therefore, other more environmentally friendly methods of suppression of plant-parasitic nematodes have been proposed. Among them, the use of plant beneficial soil bacteria, behaving as biocontrol agents against nematodes, represent a potential alternative to chemicals.

**Abstract:**

Plant-parasitic nematodes have been estimated to annually cause around US $173 billion in damage to plant crops worldwide. Moreover, with global climate change, it has been suggested that the damage to crops from nematodes is likely to increase in the future. Currently, a variety of potentially dangerous and toxic chemical agents are used to limit the damage to crops by plant-parasitic nematodes. As an alternative to chemicals and a more environmentally friendly means of decreasing nematode damage to plants, researchers have begun to examine the possible use of various soil bacteria, including plant growth-promoting bacteria (PGPB). Here, the current literature on some of the major mechanisms employed by these soil bacteria is examined. It is expected that within the next 5–10 years, as scientists continue to elaborate the mechanisms used by these bacteria, biocontrol soil bacteria will gradually replace the use of chemicals as nematicides.

## 1. Introduction

Nematodes (also called roundworms) are small (about 0.2 to 10.0 mm in length) non-segmented invertebrate that have existed for ~500 million to one billion years and are by far the most abundant animals on earth [1,2].

It has been estimated that the number of nematodes in the surface soil reaches 4.4 × 10^20^ (with a total biomass of ~0.3 gigatonnes), with a higher diffusion in sub-Arctic regions than in temperate or tropical zones [3]. Soil nematodes play a central role in the soil food web being present in all trophic levels; they regulate carbon and nutrient dynamics and modulate the microbial density [4,5]. They are believed to be efficient indicators of biological activity in soils responding to global climate changes, in particular regarding the amount of CO_2_ in the atmosphere and global warming. In this regard, the level of atmospheric CO_2_ has increased from 280 to 380 ppm over the last 150 years, and according to some mathematical models, this value is likely to double by the end of this century. Similarly, the global mean temperature is expected to undergo an increase of 1.1–6.4 °C during the same period of time [6]. With elevated CO_2_ levels, the photosynthetic rate in plants, as well as the net primary production, is expected to increase thereby inducing higher plant biomass and increased root development, followed by an enhanced amount of carbon release through root exudation [7,8]. In addition, several studies have indicated that the increased temperatures from global warming will likely cause a further increment in nematode abundance and, more consistently, in their biodiversity [9,10]. 

Soil nematodes are classified as bacterivores, fungivores, herbivores, omnivores, and predators [11]. It has been estimated that there are >1 million nematodes species mainly in the sea/ocean with a large number of nematodes in the soil or freshwater, while about 15% are hosted by animals including insects and other invertebrates, as well as domestic and wild animals and man. Approximately 3400 nematodes species behave as plant parasites (http://mrec.ifas.ufl.edu/lso/SCOUT/Nematodes.htm) [12] and cause both significant yield and economic losses in crop production [13]. However, their importance as plant pathogens is difficult to quantify as the number of nematodes found in soil is highly variable within and across terrestrial biomes and can range from dozens to thousands of individuals per 100 g soil [3]. Some species have a limited geographical distribution but can nevertheless cause widespread damage to plants in a localized area, while other species show a narrow range of plant host. About 250 species belonging to 43 genera are considered as a phytosanitary risk (i.e., a pathogenic hazard) and among them, root-knot nematodes (*Meloidogyne* spp.), as a consequence of their global distribution and high reproductive rate are the most damaging in agriculture, followed by cyst nematodes (*Heterodera* and *Globodera* spp.), root lesion nematodes (*Pratylenchus* spp.), the burrowing nematode (*Radopholus similis* Cobb), and the stem nematode (*Ditylenchus dipsaci* Filipjev) [14,15].

The nematode infection cycle begins with insertion of the stylet (a hollow mouth spear) into plant tissue. This causes serious damage to the infected plant where nematodes can increase both direct and indirect symptoms of pathogen damage. Direct symptoms include low development of the whole root system, abnormal root morphology, and enlargement of the roots. Indirect symptoms are typically related to the depletion of photo-assimilates, and the reduction of water and decreased nutrient absorption [15,16]. Nematodes are a major agricultural pathogen causing ~12.3% losses per year globally, when considering the world’s 40 major crops. Moreover, the reduction in crop yield is significantly greater in developing countries (14.6%) than in developed ones (8.8%). It has been estimated that the annual global economic losses in crop yield because of plant-parasitic nematodes in major crops is USD 173 billion, notwithstanding the phytosanitary measures that are applied to control nematodes [17]. Taking into account the changes occurring in atmospheric CO_2_ levels and global warming, there is a very real possibility that future crop yields will be dramatically reduced by the expected accompanying large increase in soil nematode populations [1]. 

**Figure 1:**
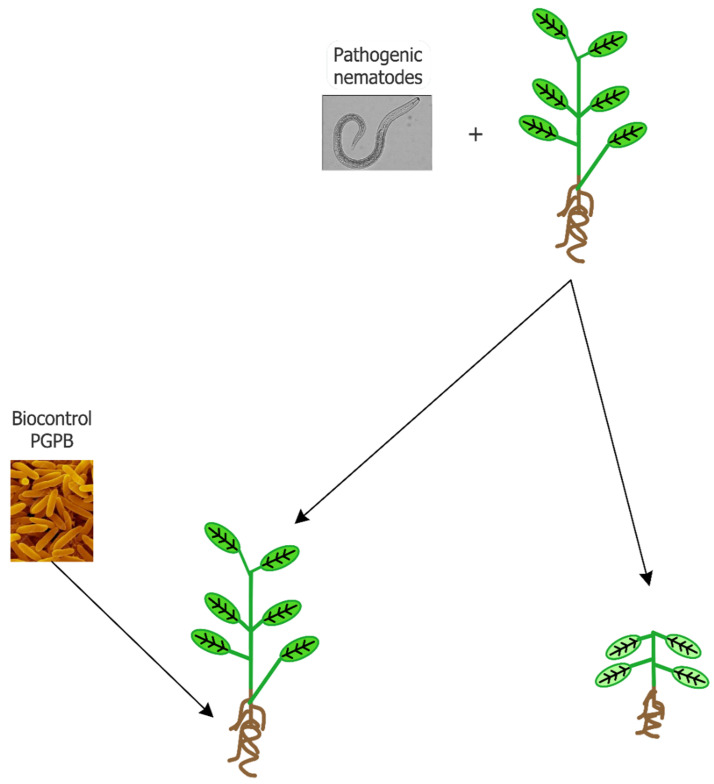
Schematic representation of the use of biocontrol plant growth-promoting bacteria (PGPB) to prevent plant damage by plant-parasitic phytopathogenic nematodes.

At present, the control of pathogenic nematodes occurs through the application of chemical nematicides that can act on respiration (isothiocyanates and halogenated aliphatic compounds), the transmission of nerve impulses (organophosphates, carbamate and abamectin), and steroid metabolism. According to their mechanism of action, nematicides can be categorized as fumigants (isothiocyanates and halogenated aliphatic compounds) and non-fumigants (organophosphates, carbamate, abamectin, and fluoroalkenyl). Fumigants affect a wide range of target organisms including fungi, bacteria, and other soil organisms, as well as seeds, so that following their application, environmental disturbance and phytotoxicity may occur. Non-fumigant molecules are formulated as either liquids or granules, both characterized by a low persistence as toxic molecules and little or no phytotoxic activity. However, they are extremely toxic to mammals and insects, having very low LD_50_ values [15]. Nematicides in the soil can also negatively affect beneficial organisms such as dung beetles, water fleas, earthworms, and nematophagous mites [18]. Moreover, nematicides are more effective when the nematodes are actively searching for a host plant in the soil. Once endoparasitic nematodes penetrate root tissues, they permanently establish themselves there, while migratory endoparasites are able to move within and between roots [19]. Unfortunately, during the internal phase of a nematode’s life cycle the efficacy of chemical nematicides is extremely low. Moreover, nematode strains that have developed a high level of resistance to chemical nematicides have emerged [20]. Based on these considerations, different strategies have been tested to better and more efficaciously control plant-parasitic nematodes in agriculture. Plant roots can release molecules (i.e., attractant compounds) in the rhizosphere that favor the colonization of roots by plant parasitic nematodes, or, alternatively, reduce the damage induced by nematodes (i.e., repellent or nematicidal compounds) [19]. The use of edible mushroom bioproducts for controlling both plant and animal parasitic nematodes has been investigated and reviewed by Castañeda-Ramirez et al. [21]. Plant growth-promoting bacteria (PGPB) is another biological tool that is useful in the control of plant-parasitic nematodes (Figure 1). 

The term PGPB refers to plant-beneficial bacteria living in the soil immediately surrounding the plant roots (rhizosphere), colonizing the root surface (rhizoplane), or living inside plant tissues (endophytic). Irrespective of their localization, PGPB can enhance plant growth and development, improve the nutritional value of edible seeds and fruits and, at the same time, protect plants from a wide range of biotic and abiotic stress [22]. This review provides an overview of recent manuscripts on the biocontrol of nematodes by PGPB. 

## 2. Mechanisms at the Base of Nematode Suppression by PGPB

**Figure 2:**
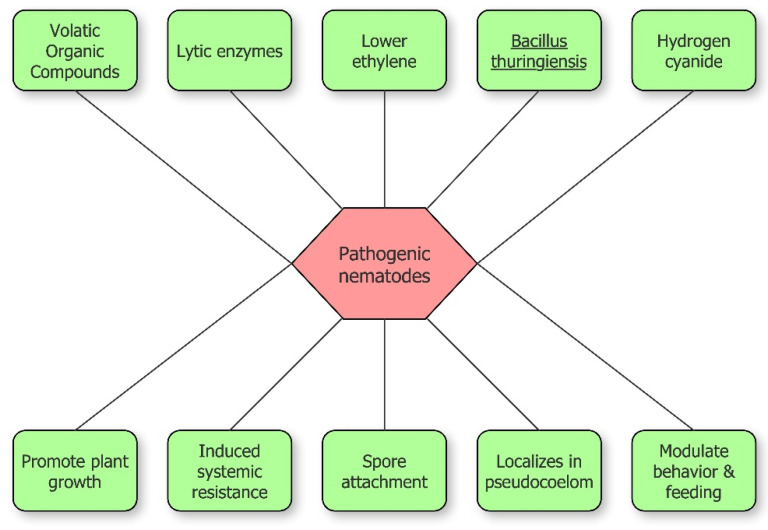
Schematic overview of bacterial inhibition of pathogenic nematodes.

Rhizobacteria inhibit plant-parasitic nematodes through different methods, both direct and indirect (Figure 2). Direct antagonism is based on the synthesis of lytic enzymes, toxic insecticidal crystal proteins, volatile compounds or parasitism. Indirect antagonism is expressed through competition for nutrients, inducing systemic resistance (ISR), or the release of molecules that modulate nematode behavior including recognition, feeding, and sex ratio [23]. The increase of plant tolerance via 1-aminocyclopropane-1-carboxylic acid (ACC) deaminase expression, lowering ethylene level in plants, is included among indirect mechanisms [1,22].

### 2.1. Lytic Enzymes

Lytic enzymes released by PGPB can induce damage both to nematode egg shells which are composed of a protein matrix and a chitin layer (an insoluble linear polymer of *N*-acetylglucosamine residues linked by β-(1,4)-glycosidic bonds) [24], as well as to the cuticle of the nematodes, that is based on a proteinaceous membrane [25]. These two structures may be cleaved by chitinases, proteases, peptidyl-peptide hydrolases, and gelatinolytic proteins.

Chitinases may be classified as either endochitinases that randomly cleave the internal portion of the chitin chain producing different *N*-acetylglucosamine monomers or exochitinases that catalyze the degradation of *N*-acetylglucosamine monomers or dimers (chitobiose) from the non-reducing end of the chitin chain [26]. Below are some examples of the use of chitinase in nematode control.

On testing a strain of *Lysobacter capsici* newly isolated from Korean soil [27] for its biocontrol activity against root-knot nematodes in tomato, this strain was found to express both chitinase and gelatinase activities. The amount of these enzymes released increased significantly following the addition of second-stage nematode eggs and juveniles of *Meloidogyne incognita* to the culture medium. The lower numbers of galls and egg masses found in tomato roots inoculated with this PGPB, compared to those occurring in uninoculated plants, was found to be a consequence of the synthesis of these enzymes.

Recently, a new chitinase-producing strain of *Chitinophaga* sp. was isolated in India [28]. This bacterial genus frequently occurs in soils with a high density of phytopathogenic fungi and plant-parasitic nematodes. Following optimization of the chitinase production by *Chitinophaga* strain S167 in liquid culture (altering pH, temperature, incubation time, and medium) the level of extracellular chitinases synthesized by this bacterial isolate increased by 48-fold so that this bacterium induced 85% mortality in second-stage juveniles of *M. incognita*.

An endophytic strain of *Bacillus cereus* (BCM2), isolated from strawberry fruits, showed high nematicidal activity against second-stage juveniles of *M. incognita*. Li et al. [29] revealed that inoculation of tomato plants with strain BCM2 increased the release from the bacterium of nematode-inhibitory molecules especially 2,4-di-tert-butylphenol and 3,3 dimethyloctane. Subsequently, it has been demonstrated that a crude protein extract of BCM2, also contained chitosanase, alkaline serine protease, and neutral protease, and induced a 100% mortality in second-stage juveniles of *M. incognita*. Electron microscopy showed that BCM2 extracellular enzymes induced nematode content leakage at the cuticle level [30].

### 2.2. Nematicidal Toxins

*Bacillus thuringiensis* (Bt) is the most studied biocontrol agent against insects, especially Lepidoptera, Coleoptera, and Diptera. Its insecticidal activity is related to the production of parasporal crystal proteins, or δ-endotoxins. Once ingested by the target insect, the protoxin is activated to form a toxin molecule in the insect’s gut by the alkaline pH and specific proteases. Then, the toxin enters the membranes of the gut epithelial cells, generates a pore through which leakage of cell contents occurs, the insect stops feeding and dies [31]. *B. thuringiensis* subspecies show insecticidal activity on different target insects according to the cry toxin they express. In addition, Bt strains producing the crystal protein families Cry5, Cry6, Cry12, Cry13, Cry14, Cry21, and Cry55 have nematicidal activity [32] against both plant and animal nematodes. In fact, the protein released by *B. thuringiensis* protein Cry5B has been proposed as a new pan-hookworm cure [33]. The efficacy of this protein is further enhanced in the presence of other physiological activities such as chitinase [34] and metalloprotease [35].

When the nematicidal effect of the Cry6Aa2 protoxin produced by a strain of *B. thuringiensis* against the root-knot nematode *Meloidogyne hapla* was studied in vitro and in soil pot conditions, it was found that Cry6Aa2 is toxic for the second-stage juvenile of *M. hapla*, and reduced egg hatch, motility, and penetration into tomato roots. These effects led to a very low galling index and mass of eggs on the host plant root, therefore increasing plant development and decreasing soil nematode amounts [36].

Complete inhibition of juvenile emergence from egg masses of *M. incognita* was obtained using a combination of parasporal crystals from 6 strains of *B. thuringiensis*. Scanning electron microscopy showed a gelatinous layer covering the egg masses of *M. incognita* after treatment with the pool of six toxins. Two of these parasporal crystals, with a very low LD_50_ (0.12 and 0.23 μg/mL of protein) were then assayed with tomato as the host plant. The data obtained show a reduced number of females in the population, decreased egg masses on the roots, and a lower root gall index compared to untreated controls or to plants treated with chemical nematicides [37].

*Xiphinema index* and *Meloidogyne ethiopica* are two of the most damaging plant-parasitic nematodes affecting grapevines in Chile. Aballay et al. [38] assessed the impact of a combination of *Bacillus* strains (6 isolates including two *B. thuringiensis* strains) and *Pseudomonas fluorescens*. The activity of these strains against *X. index* and *M. ethiopica* were previously demonstrated under in vitro and glasshouses conditions. Then, the biocontrol effect of liquid and powder formulation of this bacterial consortium was assessed on grapevine cv. Cabernet Sauvignon cultivated in a nematode infested vineyard. The results obtained indicated that a mixed bacterial inoculum was able to reduce plant damage induced by *X. index*, showing an efficiency comparable to chemical nematicides, while more varied results were observed against *M. ethiopica* [38]. A portion of the damage caused by *X. index* is a likely consequence of the plant virus that it carries. Thus, reduction of *X. index* results in less virus transmission and less damage from the virus.

### 2.3. Volatile Organic Compounds (VOCs)

Microbial VOCs are low molecular weight compounds with high vapor pressure, that can diffuse a long distance through air, soil, and water, can directly promote plant growth as well as suppress or attract pathogens, nematodes, and insects [39]. The main chemical classes of VOCs produced by microorganisms are alkanes, alkenes, alcohols, esters, ketones, terpenoids, and sulfur families [39]. It has been reported that many VOCs are considered to be infochemical molecules involved in the communication among organisms [40]. Interestingly, some of the VOCs released by *Pseudomonas* and *Serratia* behave as quorum-quenching molecules inhibiting cell-to-cell communication network, leading to a lowered synthesis of virulence and fitness factors such as antibiotics, pigments, exoenzymes, and toxins [41].

Although some VOCs that behave as chemoattractants for nematodes have been found in various *Pseudomonas* spp. [42], most of the literature is focused on the nematicidal effect of bacterial VOCs. 

*Pseudomonas chlororaphis* 449 and *Serratia proteamaculans* 94 isolated from spoiled meat, are able to synthesize 1-undecene, 2-nonanone, and 2-undecanone, and dimethyl disulfide (DMDS), respectively. Besides showing bacteriostatic/fungistatic effects on phytopathogenic *Agrobacterium tumefaciens* and *Rhizoctonia solani*, these bacterial VOCs demonstrated a killing effect on the cyanobacterium *Synechococcus*, fruit flies (*Drosophila melanogaster*), and nematodes (*Caenorhabditis elegans*). In particular, DMDS produced by S. *proteamaculans* and the ketones 2-nonanone and 2-undecanone released by *P. chlororaphis* at a concentration of 25 μmol, killed *C. elegans* after 3 days of exposure. All of the nematodes in the last juvenile form exposed to 25 μmol 2-heptanone produced by *S. proteamaculans* turned into adult nematode forms, but they were unable to produce eggs [43].

Five bacterial strains, *Pseudochrobactrum saccharolyticum*, *Wautersiella falsenii*, *Proteus hauseri*, *Arthrobacter nicotianae*, and *Achromobacter xylosoxidans,* produced a total of 53 VOCs including aldehydes, ketones, alkyls, alcohols, alkenes, esters, alkynes, acids, ethers, as well as heterocyclic and phenolic compounds. When 19 of these 53 compounds were assessed for their nematicidal activity against *C. elegans* and *M. incognita*, 7 (acetophenone, S-methyl thiobutyrate, dimethyl disulfide, ethyl 3,3-dimethylacrylate, nonan-2-one, 1-methoxy-4-methylbenzene, and butyl isovalerate) demonstrated high suppressive ability against these nematodes, with S-methyl thiobutyrate being more efficient at killing nematodes than the commercial nematicide DMDS [44].

*Bacillus* sp., *Paenibacillus* sp., and *Xanthomonas* sp. strains able to suppress the growth of the phytopathogenic fungus *Rhizoctonia solani* were assessed by both in vitro and *in planta* systems for their impact on the rice root-knot nematode *Meloidogyne graminicola.* These three biocontrol agents were lethal to *M. graminicola*; this nematode was 99% killed following three days of exposure to volatile compounds from each of the three strains. The lethal time by which 50% of the nematode population was dead ranged from 1.56 to 2.25 h, according to the bacterial strain considered [45].

### 2.4. Bacterial Nematode Hyperparasitism: Pasteuria and Its Influence on Nematode Fertility

Members of the *Pasteuria* genus are Gram positive, dichotomously branched spore forming bacteria belonging to the *Firmicutes* phylum. *Pasteuria* spp. are known for the ability to suppress plant pathogenic nematodes through two mechanisms. The first one is based on spore attachment to the surface of nematodes in the juvenile phase; this leads to inhibited movement toward the plant root. Second, *Pasteuria* cells penetrate the nematodes and localize, with a high density, inside the pseudocoelom affecting embryogenetic processes and impairing host reproduction. The life cycle of *P. penetrans* accounts for three stages: spore attachment and germination, exponential growth with rhizoid production and generation of new spores, occurring when the root-knot nematodes in the second juvenile phase establish permanent feeding sites in their plant hosts [46]. Then, *Pasteuria* cells replicate inside the nematode, killing it and transforming what was a female in a “bag of endospore” [47]. Nematode biocontrol occurs when endospores of *P. penetrans* reach a density of ~10^4^ to 10^5^ endospores/g soil [48]. *Pasteuria* spp. shows different levels of specificity for the host that can vary from species to populations [49]. The susceptibility of the nematode cuticle to endospore adhesion is affected by the release of root exudates from a host plant leading to hyperparasitic recruitment [50]. On the other hand, the weakened cuticle of the juvenile nematodes is lost during the internal colonization of the roots so that it behaves as an elicitor of plant immune responses [51].

To determine if *P. penetrans* was present at a density sufficient to reduce *M. javanica* in soil from a sugarcane field showing a high level of second-stage juveniles of root-knot nematodes, pot experiments were performed. The results indicated that root-knot nematode populations increased when inoculated in sterilized soils, while the nematode amount was reduced by 96% in untreated soil. Sugarcane was cultivated in soil supplemented with different endospore concentrations. The highest value of endospores of *P. penetrans*/g soil, corresponded to an 80% reduction of nematodes. Simultaneously, the severity of root galling and the number of nematode eggs produced per plant decreased as the endospore concentration increased [52].

Among the three phases of the *Pasteuria* life cycle, endospore binding to the cuticle of second-stage nematode juveniles is a critical step of the hyperparasitic relationship. Recently, Phani et al. [47] shed light on the early transcriptional response of *M. incognita* eight hours after endospore attachment. RNA was subsequently extracted from about 20,000 *M. incognita* non-encumbered and endospore encumbered nematodes in the second juvenile phase. Of 52,485 transcripts, 582 were differentially expressed between the two groups: 229 were up-regulated and 353 were down-regulated. The down-regulated genes were mainly involved in nematode protein synthesis, innate immunity, signaling, stress responses, endospore attachment processes, and post-attachment behavioral modification. Analysis of 15 transcripts revealed that endospore attachment of the cuticle nematode is regulated by fructose bisphosphate aldolase, glucosyl transferase, aspartic protease, and ubiquitin.

### 2.5. Induced Systemic Resistance

Some PGPB can improve plant health through the stimulation of plant defense responses, i.e., through ISR. This stimulation is mediated by microbial elicitors such as VOCs, siderophores, flagellin and lypopolisaccharide (LPS) all of which trigger ISR by means of a variety of plant hormones such as jasmonates, ethylene, auxin, and nitrogen oxide [53]. Systemic resistance induced by PGPB against nematodes was observed for the first time in tomato infested by *M. incognita* and *M. arenaria* [54].

Siddiqui and Shaukat [55] investigated the ISR mechanisms in tomato stimulated by *Pseudomonas aeruginosa* strain IE-6S+ and *P. fluorescens* strain CHA0 in response to *M. javanica*. First, the ability of the two rhizobacteria to elicit ISR against nematodes was demonstrated in split-root-trials, where the root of a single plant was distributed into two pots, each containing 350 g of soil. After inoculation of strains IE-6S+ and CHA0 (each in a separated pot) nematode penetration into the other part of the root was reduced by 42% and 29%, respectively. Interestingly, while the cell density of strain CHA0 was high during early tomato development, IE-6S+ showed a high rhizospheric colonization rate at late plant developmental stages and was able to penetrate plant tissue. Since jasmonates are triggered by ISR, Soler et al. [56] assessed the effect of methyljasmonate on *Rotylenchulus reniformis* suppression in pineapple cv. Smooth Cayenne and MD-2. The results showed a 67% reduction of the nematode population with cv. MD-2, while the level of nematodes remained unaffected following the treatment of cv. Smooth Cayenne. When applied to pineapple cv. MD-2, grown in a split root system, methyljasmonate induced a transient stress on the plants, followed by an increased enzymatic activity expression of lipoxygenase and superoxide dismutase, and to a lesser extent peroxidase phenylalanine ammonia lyase and chitinase; suggesting that ISR activation against *R*. *reniformis* in pineapple can differ according to the plant cultivar [56].

When the ability of *Pseudomonas aeruginosa* and *Burkholderia gladioli* to promote the growth of tomato plants infested by *M. incognita* was assessed [57], nematode infection negatively impacted plant growth and caused the accumulation of superoxide anion, hydrogen peroxide, and malondialdehyde in plant tissues while inoculation with the two bacterial strains promoted plant growth and significantly reduced the number of galls. Plants infested by nematodes showed an increased amount of antioxidative enzymes (superoxide dismutase, peroxidase, catalase, glutathione reductase, glutathione transferase, ascorbate peroxidase, glutathione peroxidase, dehydroascorbate reductase and polyphenol oxidase). In infested plants inoculated with the two PGPB, the concentrations of these enzymes was even higher. Thus, the combination of these two bacterial strains was able to promote plant growth and to increase plant resistance to nematode infection by modulating the plant’s antioxidative potential, an effect thought to be, but not proven to be related to ISR.

Six compounds produced by *Bacillus simplex* Sneb545 in soybean infested by *Heterodera glycines* were classified as elicitors of ISR [58]. These molecules, identified through H-1 NMR and C-13 NMR as cyclic(Pro-Tyr), cyclic(Val-Pro), cyclic(Leu-Pro), uracil, phenylalanine, and tryptophan, were able to postpone the development of the plant-parasitic nematode on soybean roots. Moreover, a low number of nematodes occurred in seedlings treated with cyclic(Pro-Tyr), cyclic(Val-Pro), and tryptophan; this was shown to be a consequence of the expression of defense genes involved in the salicylic acid and jasmonate pathways against *H. glycines*. Moreover, these cyclic dipeptides behave as a signal molecule activating the biosensors of *N*-acyl-L-homoserine lactone and are involved in antibacterial, antifungal, antiviral and antitumor activities [58]. Phenylalanine is a salicylic acid (SA) precursor and the role of SA as a signal for modulating pathogenensis-related protein synthesis is well known [59]. Similarly, the amino acid tryptophan is involved in the synthesis of various secondary metabolites including auxins, phytoalexins, and alkaloids, that enhance plant development and stimulate resistance to the attack by phytopathogenic organisms [60].

### 2.6. Modulation of Nematode Behavior, Feeding, and Movement 

Bacterial cells represent the main source of nutrients for bacterial-feeding nematodes in soils. However, these nematodes have specific “tastes” and are able to judge if the bacterial prey is beneficial before feeding. For example, *C. elegans* can distinguish bacteria that act as nematode pathogens, such as *Pseudomonas aeruginosa* and *Serratia marcescens*, and avoid them. This behavior appears to be mediated by the recognition of molecules of bacterial origin such as CO_2_, indole, and the quorum-sensing autoinducer *N*-acyl-homoserine lactones. In fact, *C. elegans* can sense the autoinducers produced by many Gram-negative bacteria, whose fitness and virulence factors are under the regulation of quorum-sensing systems [61].

When cultivated with two fast growing (*Pseudomonas fluorescens* Y1 and *Escherichia coli* OP50) and three slow growing (*Bacillus amyloliquefaciens* JX1, *Variovorax* sp. JX14, *Bacillus megaterium* JX15) bacterial strains, *C. elegans* showed a marked feeding preference for the fast growing bacterial cells. This feeding behavior, probably induced by a high respiration rate and CO_2_ emitted as an attractant for the nematode, leads to different consequences in terms of longevity and reproduction efficiency. Nematodes feeding on fast growing bacterial species produced more offspring, but had a shorter lifespan, while those feeding on the slow growing bacterial strains had increased lifespans and reduced brood size. The data suggest that the metabolism of fast growing bacteria affects the behavior of *C. elegans* by attracting nematodes. Consequently, once they reach the nematode’s gut, the substrate utilization rate becomes very fast and a reduction in longevity occurs. Slow-growing bacteria are not the preferential food for nematodes. As a result, the longevity of the nematodes increase, but their fertility decreases. Thus, the preferred food may not be the most beneficial for *C. elegans*, meaning that nematodes need to find an equilibrium between feeding on preferred vs. beneficial bacteria [62].

Besides being affected by the respiration rate of soil bacteria, *C. elegans* behavior is affected by indole in both Gram positive and negative bacterial species. Indole behaves as a signal molecule for different bacterial activities such as modulation of endospore formation, plasmid stability, cell replication, antibiotic resistance, and expression of virulence factors such as biofilm formation [63]. *C. elegans* is attracted by indole-producing bacteria and avoids pathogenic bacteria unable to synthesize this metabolite. Moreover, the egg laying rate in *C. elegans* was enhanced by indole-producing bacteria, and reduced by the bacterial pathogens [64]. The pathogen avoidance behavior observed in *C. elegans* is fundamental to ensure its survival in the environment, thus lowering the risk of infection. Ingestion of bacterial cells representing a beneficial food source leads to the development of learned attraction, while infection by pathogenic bacteria, stimulates a cascade of reactions due to innate immunity, generating stress responses and host damage, leading to an aversive behavior [65].

Several soil bacteria produce biofilms through the release of signal molecules and quorum- sensing regulation. Among them, *Pseudomonas aeruginosa* is able to form biofilm embedded in an exopolysaccharide matrix that gives protection to the members of the biofilm from external environmental factors. Inside the biofilm, the synthesis of quorum-sensing regulated virulence factors occurs; among them, the siderophore pyoverdine kills *C. elegans* after being internalized into the nematode gut [66], while cyanide induces nematode paralysis [67]. After a short exposure to *P. aeruginosa* cells, *C. elegans* learns to avoid it when subsequently exposed [68]. 

Using a *P. aeruginosa* mutant library, unable to synthesize different biofilm component, the key role of the exopolysaccharide Psl, is identified. Pls hampers *C. elegans* movement and induces a nematode behavior called “quagmire” phenotype. As a result, *C. elegans* remains entrapped in the biofilm becoming unable to move far from negative stimuli or to reach favorable areas for grazing in the biofilm. Overall, these results suggest a motility impairment by Pls and emphasize the relevance of this factor in the relationship between prey and predator [69].

### 2.7. Alleviation of Nematode Induced Plant Stress through 1-Aminocyclopropane-1-Carboxylate (ACC) Deaminase 

Nematode attack is a stressful condition for plants, and as happens when plants face biotic or abiotic stress, the levels of the phytohormone ethylene in plants increase generating two peaks. The first (small) ethylene peak induces the synthesis of defensive genes. If the stressful condition persists or becomes more intense, then a second, larger peak of ethylene occurs causing the plant to exhibit symptoms and, possibly, die. Some PGPB strains are able to synthesize the enzyme 1-aminocyclopropane-1-carboxylate (ACC) deaminase that cleaves ACC, the immediate precursor of ethylene in plants, producing ammonia and alpha-ketobutyrate [70]. Therefore, ACC deaminase, modulating ethylene levels, represents one of the most relevant bacterial physiological traits by which a PGPB is able to support plant growth under stressful conditions, preventing the amount of ethylene synthesized by the plant from reaching levels that are deleterious for plant growth [71,72]. ACC deaminase genes are common in soil bacteria and plant inoculation with bacteria able to produce this enzyme increases plant tolerance to heavy metals, salinity, drought, organic pollutants, phytoplasma infection, and pathogenic fungi colonization [73].

The effectiveness of *P. putida* UW4, a model PGPB strain able to produce ACC deaminase was used as a control agent for pine wilt disease (abbreviation PWD) induced by the pinewood nematode *Bursaphelenchus xylophilus* [74]. Symptoms of PWD include reduced flux of the oleoresin inside the tree, inhibition of photosynthesis leading to browning/reddening of the needles and, reduction of the xilematic transport of water, inducing wilting. The disease is transmitted among plants by the insect vector *Monochamus* spp. [75]. Seedlings of *Pinus pinaster* were inoculated with or without strain UW4 and its mutant AcdS^–^, lacking the ability to synthesize ACC deaminase, and cultivated in the presence of the nematode. Symptoms of PWD were significantly reduced by inoculation with wild-type *P. putida* UW4, while seedlings infested with the bacterial mutant showed the expected symptoms of PWD. These data indicate that ACC deaminase is involved in plant protection against nematode damage. Moreover, seedlings inoculated with *P. putida* UW4 were colonized by nematodes to a lesser extent compared to either uninoculated plants or plants inoculated with the bacterial mutant. *P. putida* UW4 did not show any in vitro nematicidal effects on *B. xylophilus*, indicating that this bacterial strain is able to boost the plant defenses against the nematode without having a direct effect on the nematode population [74].

## 3. Conclusions

Intensive agriculture leads to high crop yields but can also cause dramatic environmental impacts. In particular, the use of many pesticides and fertilizers has caused significant damage both to the environment and to human health. About 3400 nematodes species are able to parasitize plants leading to reduced crop yield and economic losses. In parallel, the demand for food increases with an increasing human population. For these reasons, alternative more environmentally friendly strategies are being sought to control plant pathogens. In this context, PGPB offer a healthy and effective but not yet fully appreciated opportunity. As the knowledge of PGPB increases, it is expected that researchers will develop techniques for improving the performance of these bacteria in suppressing the growth of phytopathogenic nematodes. This should allow us to realize the goal of more efficient and environmentally friendly sustainable agriculture.
